# Indeterminate QuantiFERON Results in Pediatric Kawasaki Disease: Inflammatory Predictors and Diagnostic Implications

**DOI:** 10.7759/cureus.88042

**Published:** 2025-07-15

**Authors:** Koji Yokoyama, Mitsukazu Mamada

**Affiliations:** 1 Department of Pediatrics, Japanese Red Cross Wakayama Medical Center, Wakayama, JPN

**Keywords:** indeterminate, interferon-gamma release assay (igra), intermediate positive for t-spot, kawasaki disease (kd), tuberculosis infection

## Abstract

Introduction

Interferon-gamma release assays (IGRAs), such as the QuantiFERON®-TB Gold Plus (QFT), are widely used for tuberculosis (TB) screening in children. However, indeterminate QFT results remain a diagnostic challenge, particularly in patients with Kawasaki disease (KD), in whom systemic inflammation may transiently impair T-cell function. This study aimed to identify clinical and laboratory factors associated with indeterminate QFT results in pediatric patients, with a focus on KD.

Methods

We retrospectively analyzed 147 pediatric QFT tests performed at a tertiary medical center in Japan between September 2019 and May 2025. Clinical characteristics and laboratory parameters were compared between patients with indeterminate and negative QFT results. Subgroup analyses were conducted for children with KD.

Results

Among the 147 pediatric cases, 30 (20.4%) yielded indeterminate QFT results, 24 (80%) of which involved KD. In the KD subgroup, the indeterminate group had significantly higher C-reactive protein levels (median 5.65 vs. 3.21 mg/dL; p=0.016) and lower serum albumin levels (2.75 vs. 2.90 g/dL; p=0.013) compared to the negative group. No significant differences were observed in other laboratory parameters.

Conclusion

This study suggests that QFT may yield indeterminate results in pediatric KD during the acute inflammatory phase, potentially reflecting transient inflammation-induced T-cell suppression. Clinicians should consider the timing of IGRA testing, alternative assays such as T-SPOT.TB, and adjunctive diagnostic tools when screening for TB in this population.

## Introduction

Interferon-gamma release assays (IGRAs), such as the QuantiFERON®-TB Gold Plus (QFT; Qiagen, Hilden, Germany), are widely used in tuberculosis (TB) screening, especially in children requiring immunosuppressive therapy. While TB remains a relevant health issue, this study focuses on the diagnostic behavior of QFT in pediatric patients with inflammatory conditions such as Kawasaki disease (KD). While the tuberculin skin test (TST) is used traditionally for TB screening, IGRAs are valuable alternatives because of their higher specificity and logistical advantages, particularly in Bacillus Calmette-Guérin (BCG)-vaccinated populations. However, it is important to note that QFT testing typically requires a blood sample of approximately 10 mL, which may pose a challenge in young pediatric patients, especially during the acute phase of illness. The QFT is one of the most widely used IGRAs, offering high sensitivity and specificity for *Mycobacterium tuberculosis* infection (including both latent and active forms), with validated performance even in pediatric cases [1]; however, indeterminate results pose a significant diagnostic challenge as they can complicate patient management and delay necessary interventions. This issue is more pronounced in young children, whose immature immune systems, susceptibility to acute inflammation, and nutritional vulnerabilities may compromise IGRA reliability. KD, a common type of pediatric vasculitis, is sometimes treated with corticosteroids or biologics when intravenous immunoglobulin (IVIG) is insufficient [2]. TB screening is recommended prior to initiating corticosteroids or biologics in cases of IVIG-resistant KD; however, the need for prompt treatment often precedes the availability of definitive screening results, complicating treatment decisions. The acute inflammatory milieu in KD is linked to impaired IGRA performance, as demonstrated by recent large-scale studies reporting high rates of indeterminate QFT results [3]. To date, there is no standardized approach to interpreting IGRA results in the setting of acute systemic inflammation, and clinicians often face diagnostic uncertainty when managing patients with indeterminate findings [4]. In countries such as Japan, where the incidence of KD is high and universal BCG vaccination is implemented [5,6], the reliability of QFT in pediatric inflammatory settings warrants particular attention. While several large-cohort studies demonstrate increased rates of indeterminate QFT results in KD cases, few have undertaken a systematic examination of the immunologic or clinical predictors of such outcomes in pediatric populations [7]. Therefore, understanding the factors associated with indeterminate QFT results in this population has important implications for clinical decision-making, particularly in IVIG-resistant KD cases where immunosuppressive therapy may be urgently required. We initially sought to assess QFT utility for TB screening in KD patients prior to immunosuppressive therapy. However, unexpectedly high rates of indeterminate results led us to investigate clinical and laboratory factors associated with QFT performance in this setting. This study aimed to identify clinical and inflammatory predictors of indeterminate QFT results in pediatric KD patients.

## Materials and methods

This retrospective, single-center study enrolled patients who underwent QFT testing at the Japanese Red Cross Wakayama Medical Center, located in Wakayama City, Wakayama Prefecture, Japan, from September 2019 to May 2025. QFT testing was typically performed at the time of initial evaluation and prior to the administration of IVIG, especially in cases where second-line immunosuppressive therapy (e.g., corticosteroids or cyclosporine) was being considered. However, the exact timing of QFT testing relative to disease onset or treatment was not standardized and varied between patients depending on clinical urgency and logistical factors. The decision to perform QFT was made at the discretion of the treating physician based on clinical judgment and institutional practice. Blood samples were processed on the same day, and QFT assays were performed in accordance with the manufacturer's instructions (QuantiFERON®-TB Gold Plus, Qiagen), including incubation and interpretation protocols. Both adult and pediatric cohorts were examined, and subgroup analysis was performed for KD patients. As this was the only positive case, it was excluded from analysis due to the lack of a sufficient number of cases for meaningful comparison. Inflammatory, coagulation, and biochemical parameters were analyzed as variables. Continuous variables were compared using the Mann-Whitney U test, a nonparametric test that does not require an assumption of normality. Categorical variables were analyzed using the chi-squared test or Fisher's exact test, as appropriate. A p-value of <0.05 was considered statistically significant. In this cohort, QFT was used both as a pre-immunosuppressive screening tool and as part of differential diagnosis for suspected TB infection, reflecting its broad utility in pediatric clinical practice. Statistical analysis was conducted using GraphPad Prism version 10.4.2 (GraphPad Software, San Diego, California, United States). The Ethics Committee of the Japanese Red Cross Wakayama Medical Center approved this retrospective study (approval number: 1467), including a waiver of informed consent.

## Results

Adult cohort

**Figure 1:**
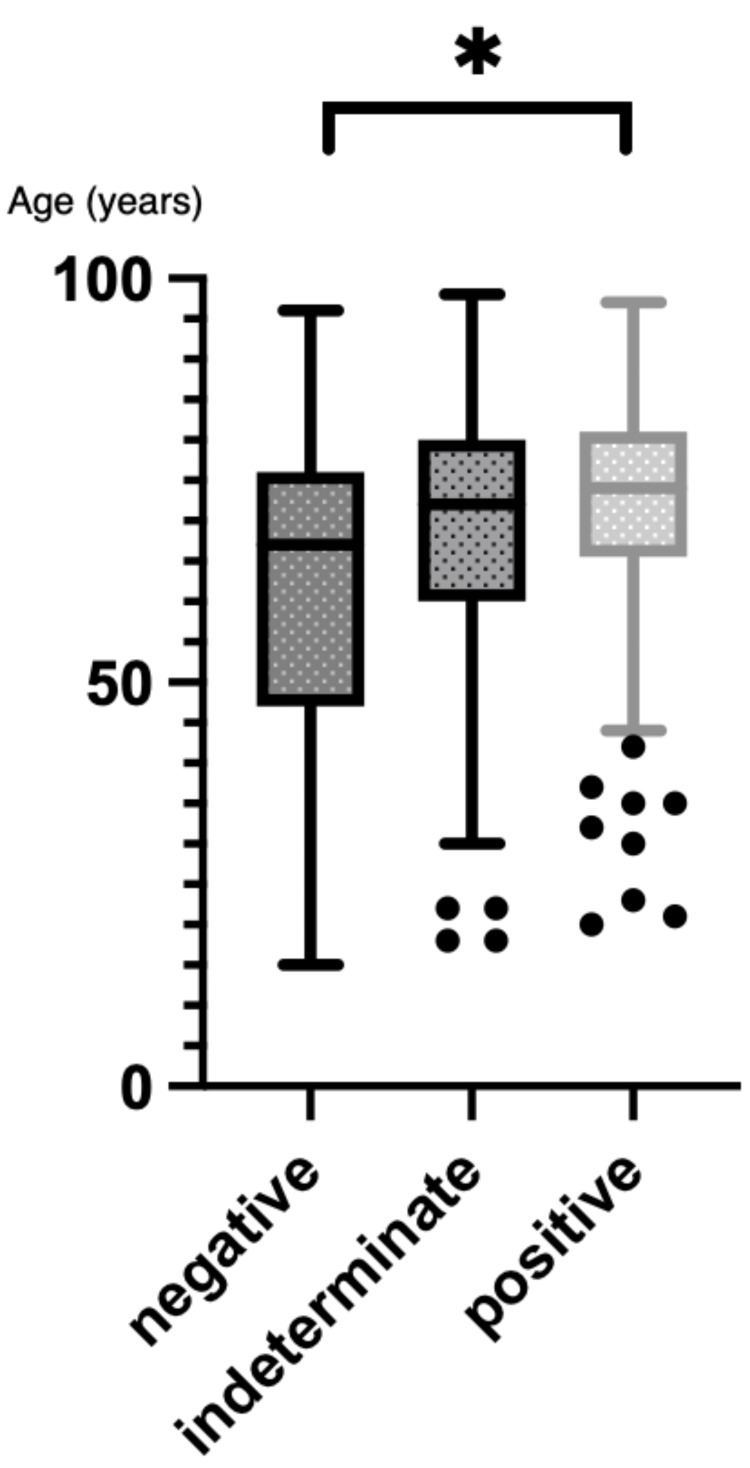
Age distribution of adult patients according to QFT results Box-and-whisker plots show median ages and IQR for patients with negative, positive, and indeterminate QFT results. Error bars represent 95% confidence intervals. Total patients: 6,045 (5,561 negative, 351 positive, and 133 indeterminate). QFT tests were conducted from 2019 and 2025. QFT: QuantiFERON®-TB Gold Plus; IQR: interquartile range

Among 6,045 adult QFT tests, 5,561 (92%) were negative, 351 (5.8%) were positive, and 133 (2.2%) were indeterminate. The positive group (median age 72; IQR: 68-80) was significantly older than the negative group (median age 60; IQR: 48-72; p=0.011). There was no significant age difference between the negative and indeterminate groups (median age 65; IQR: 56-79; p=0.144; Figure 1).

**Figure 2:**
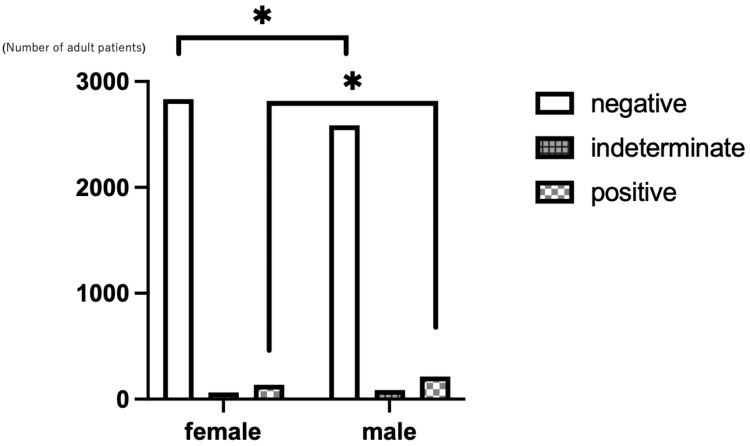
Sex distribution by QFT result in adult patients Bar graph showing the number of male and female patients in each QFT result category (negative, positive, and indeterminate). Total patients: 6,045 (3,007 males and 3,038 females). QFT tests were conducted from 2019 to 2025. QFT: QuantiFERON®-TB Gold Plus

There was a significant difference in sex distribution among the groups (p<0.001), with females predominating in the negative group (52.3%) and males in the positive group (61.1%; Figure 2).

Among the 133 indeterminate adult cases, 60 (45.1%) were receiving corticosteroids, and nine (6.8%) were treated with other immunosuppressive agents. Of the 64 indeterminate patients not receiving these therapies, the major underlying conditions were renal disease (28 cases), collagen vascular disease (11 cases), or malignancy (10 cases; Table 1).

**Table 1:** Clinical characteristics of adult patients with indeterminate QFT results ※The infectious disease group does not include tuberculosis or nontuberculous mycobacterial infections. Among 133 adult cases with indeterminate QFT results, 60 (45.1%) were receiving corticosteroids, and nine (6.8%) were treated with other immunosuppressive agents. Among the remaining patients, common underlying conditions included kidney disease (21.1%), rheumatic diseases (8.3%), malignant disease (6.8%), and infectious diseases (5.3%). Tuberculosis and nontuberculous mycobacterial infections were categorized separately (3.8%) and not included in the general infectious disease group. QFT: QuantiFERON®-TB Gold Plus; n: number; %: percentage

Clinical characteristics	n (%)
Corticosteroid use	60 (45.1)
Immunosuppressive medication	9 (6.8)
Kidney disease	28 (21.1)
Rheumatic diseases	11 (8.3)
Malignant disease	9 (6.8)
Infectious disease※	7 (5.3)
Tuberculosis/nontuberculous mycobacteria	5 (3.8)
Others	4 (3)

Pediatric cohort

Among 147 pediatric QFT tests, 116 (78.9%) were negative, one (0.7%) was positive, and 30 (20.4%) were indeterminate. The single positive case involved a 56-month-old child who developed latent tuberculosis infection (LTBI) after recent travel from China and was excluded from statistical comparison due to its distinct epidemiological and clinical background. Further details regarding this case are provided in the Appendices [8]. There were no significant differences in age (p=0.22) or sex distribution (p=0.40) between the negative and indeterminate groups. Of the 30 pediatric patients, two had juvenile idiopathic arthritis (JIA; treated with corticosteroids), two had idiopathic nephrotic syndrome, one had a kidney abscess, and one had acute disseminated encephalomyelitis (ADEM). The remaining 24 cases (80%) were KD. Corticosteroids were not used in cases of nephrotic syndrome, kidney abscess, or ADEM (Table 2). 

**Table 2:** Clinical characteristics of pediatric patients with indeterminate QFT results Among 29 pediatric patients with indeterminate QFT results, the majority (n=23; 79.3%) were diagnosed with Kawasaki disease. Other diagnoses included juvenile idiopathic arthritis treated with corticosteroids (n=2), idiopathic nephrotic syndrome (n=2), kidney abscess (n=1), and ADEM (n=1). Immunosuppressive therapy was not used in the cases of nephrotic syndrome, kidney abscess, or ADEM. QFT: QuantiFERON®-TB Gold Plus; ADEM: acute disseminated encephalomyelitis; n: number; %: percentage

Clinical diagnosis	n (%)
Juvenile idiopathic arthritis with corticosteroids	2 (6.9)
Idiopathic nephrotic syndrome	2 (6.9)
Kidney abscess	1 (3.4)
ADEM	1 (3.4)
Kawasaki disease	23 (79.3)

Subgroup analysis of non-KD cases was not performed because of the low number of such cases.

Subgroup analysis: Kawasaki disease

**Figure 3:**
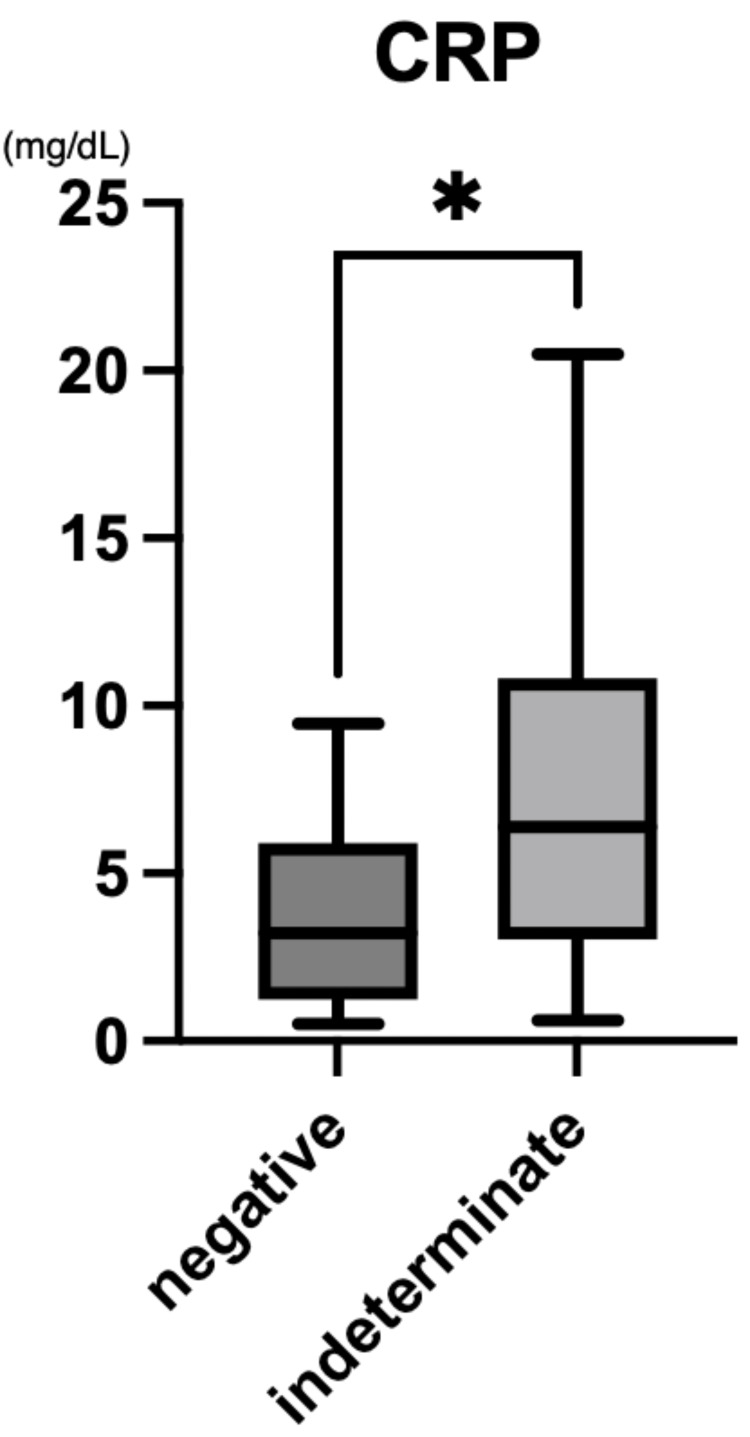
CRP levels in QFT-indeterminate and QFT-negative patients with Kawasaki disease Box-and-whisker plots show median values and IQR. CRP was measured in mg/dL. A statistically significant difference was observed between the two groups (p=0.016). Total KD patients analyzed: 40 (24 indeterminate, 16 negative). QFT tests were conducted between 2019 and 2025. QFT: QuantiFERON®-TB Gold Plus; CRP: C-reactive protein; IQR: interquartile range

Among the 41 KD patients who underwent QFT testing, 24 were indeterminate and 16 were negative. The indeterminate group had significantly higher C-reactive protein (CRP) levels (median 5.65 mg/dL; IQR: 3.16-10.41) than the negative group (median 3.21 mg/dL; IQR: 1.48-5.39; p=0.016; Figure 3).

**Figure 4:**
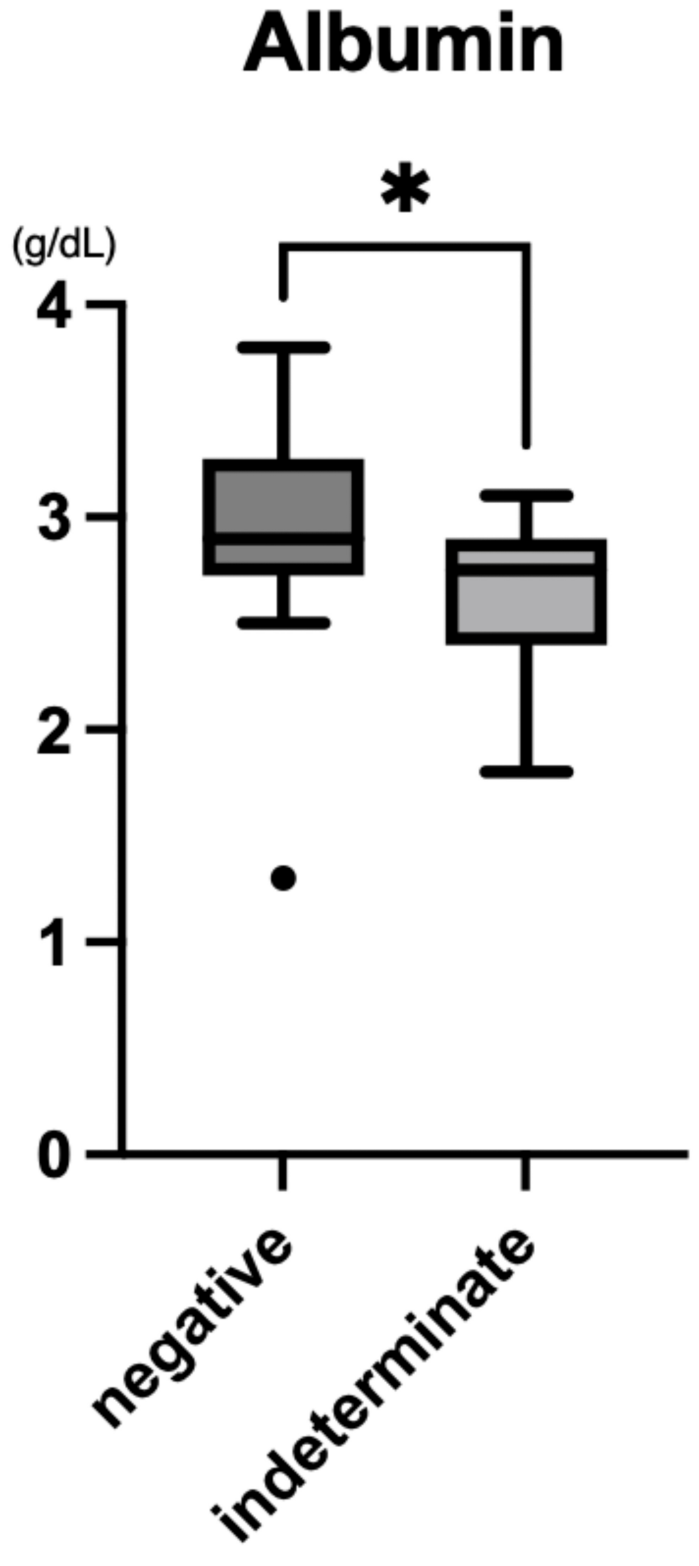
Serum albumin levels in QFT-indeterminate and QFT-negative patients with Kawasaki disease Box-and-whisker plots show median values and IQR. Albumin was measured in g/dL. A statistically significant difference was observed between the two groups (p=0.013). Total KD patients analyzed: 40 (24 indeterminate, 16 negative). QFT tests were conducted between 2019 and 2025. QFT: QuantiFERON®-TB Gold Plus; IQR: interquartile range

Serum albumin levels were also significantly lower in the indeterminate group (median 2.75 g/dL; IQR: 2.40-2.90) than in the negative group (median 2.90 g/dL; IQR: 2.77-3.23; p=0.013; Figure 4).

There were no significant differences between the groups with respect to other laboratory parameters, including inflammatory, coagulation, hepatic, and renal markers. A detailed list of laboratory variables assessed is summarized in Table 3. 

**Table 3:** Laboratory parameters assessed in KD patients undergoing QFT testing Variables include inflammatory (e.g., CRP, WBC), hepatic (e.g., AST, ALT), renal (e.g., BUN, creatinine), coagulation (e.g., PT, fibrinogen, D-dimer), and hematologic markers. Units and reference ranges are based on institutional standards or pediatric norms. WBC: white blood cell count; CRP: C-reactive protein; AST: aspartate aminotransferase; ALT: alanine aminotransferase; BUN: blood urea nitrogen; PT: prothrombin time; QFT: QuantiFERON®-TB Gold Plus; KD: Kawasaki disease

Parameter	Unit	Reference range (typical)
WBC	×10³/μL	4.0-8.0
Hemoglobin	g/dL	11.5-14.5
Platelet count	×10⁴/μL	15-45
CRP	mg/dL	<0.3
Serum albumin	g/dL	3.8-5.0
AST	U/L	<40
ALT	U/L	<35
BUN	mg/dL	7-20
Creatinine	mg/dL	0.2-0.8
Sodium	mmol/L	135-145
Potassium	mmol/L	3.5-5.0
PT	sec	10-13
Fibrinogen	mg/dL	200-400
D-dimer	μg/mL	<1.0

## Discussion

In this study, we found a notably high frequency of indeterminate QFT results among pediatric patients with KD. Within this subgroup, elevated CRP levels (Figure 3) and decreased serum albumin concentrations (Figure 4) were significantly associated with indeterminate outcomes. These findings suggest that acute systemic inflammation during the active phase of KD may transiently suppress T-cell responsiveness, thereby impairing IGRA performance. Unlike patients with known immunosuppression or critical illness [4], the immune dysfunction in KD appears to be transient and inflammation-driven. This distinction has important diagnostic implications, particularly when TB screening is required before initiating immunosuppressive therapy [4,9].

Interpretation of inflammation-related mechanisms

In the acute phase of KD, hypoalbuminemia likely reflects increased vascular permeability rather than malnutrition, a hallmark of the disease's inflammatory pathology [10]. This phenomenon is driven by interleukin-6-mediated signaling and release of vascular endothelial growth factor and angiopoietin-like proteins, which contribute to albumin leakage and elevated CRP levels [10,11]. These inflammatory mediators may suppress T-cell function transiently and inhibit mitogen-induced production of interferon-gamma (IFN-γ), resulting in an indeterminate IGRA response [4,9]. Comparable mechanisms have been documented in patients within intensive care units and in individuals with systemic infections, providing further support for inflammation-induced T-cell hyporesponsiveness [4,9]. However, we acknowledge that our study did not include direct immunological measurements, such as T-cell function assays or cytokine profiling. As such, the proposed mechanistic explanations remain speculative and require validation through future studies incorporating targeted immunological analyses.

Implications for clinical practice and diagnostic strategies

These diagnostic limitations are particularly relevant when immunosuppressive therapy must be initiated before TB infection can be excluded definitively. In such cases, repeating IGRA testing after the resolution of the acute-phase response or selecting an alternative assay such as the T-SPOT.TB test which has been reported to be less susceptible to systemic inflammation due to its use of standardized cell counts and ELISPOT-based detection [12,13] may be considered as a future direction. However, we did not perform comparative testing in this study; therefore, this recommendation remains speculative. Based on these observations and previously published literature, we suggest several hypothesis-generating strategies that may improve TB screening accuracy in the context of acute KD, although these remain unvalidated in our study and warrant future investigation. First, the timing of IGRA testing should be optimized in accordance with the inflammatory state, particularly in IVIG-resistant cases that require urgent treatment [3,11]. Second, clinicians should consider alternative approaches when an indeterminate result is anticipated. The T-SPOT.TB assay has demonstrated resilience against inflammation-related interference [14,15], and adjunctive tools such as chest imaging and clinical risk stratification further support decision-making [16]. Microbiological testing methods, including nucleic acid amplification tests and gastric aspirate cultures, may also aid diagnosis in high-risk or symptomatic cases [17,18]. Lastly, the development of KD-specific diagnostic algorithms incorporating inflammatory biomarkers and treatment urgency is warranted to guide clinicians through indeterminate results. Similar strategies may prove useful in other pediatric conditions characterized by acute systemic inflammation and early immunosuppressive therapy, such as JIA [19,20]. Our findings underscore the importance of recognizing that systemic inflammation in KD can impair the performance of IGRA-based TB screening. In clinical practice, this means that a single indeterminate QFT result obtained during the acute phase of KD may not reliably reflect TB infection status. Clinicians should consider repeating the test during convalescence or integrating alternative assessments (e.g., history, imaging) before delaying immunosuppressive treatment based solely on indeterminate results. While CRP and albumin were significantly associated with indeterminate QFT outcomes in our cohort, their predictive utility has not been formally tested. Future studies are needed to evaluate whether these biomarkers can be incorporated into validated clinical decision tools.

This study has several limitations. First, its retrospective, single-center design and relatively small sample size may limit generalizability. Second, the timing of QFT testing in relation to disease phase or IVIG administration was not standardized, potentially affecting the results. Third, follow-up testing was not performed systematically in cases with indeterminate results, and clinical outcomes were not assessed. In addition, the study did not include direct comparisons with alternative assays such as T-SPOT.TB, nor did it assess imaging or microbiological diagnostic modalities. Although we discussed the potential advantages of T-SPOT.TB, its performance was not evaluated in this study; thus, any recommendations remain speculative. Furthermore, while our findings suggest inflammation-induced suppression of T-cell responsiveness, this mechanism remains hypothetical in the absence of direct immunological measurements. Lastly, the relatively small number of pediatric KD cases undergoing QFT testing may, in part, reflect ethical and practical concerns regarding blood sampling in young children. The QFT assay requires multiple milliliters of blood, which may limit its applicability in infants and toddlers where sampling volume is constrained. These limitations highlight the need for future prospective studies with larger cohorts, standardized testing protocols, immunological correlates, and outcome data to clarify the clinical utility of IGRAs in acute pediatric inflammatory diseases. Additionally, this study was exploratory in nature and should be considered hypothesis-generating. Future multicenter, prospective studies with follow-up of indeterminate cases will be needed to validate these preliminary findings and assess their clinical implications.

## Conclusions

This study suggests that QFT may yield indeterminate results in children with KD during the acute inflammatory phase, which may reflect transient inflammation-induced T-cell suppression. Elevated CRP and decreased albumin levels were associated with indeterminate results, likely reflecting transient inflammation-induced T-cell suppression. To improve TB risk assessments in this population, clinicians should consider the timing of IGRA testing, alternative assays such as T-SPOT.TB, and adjunctive diagnostic tools. Furthermore, the development of KD-specific diagnostic algorithms incorporating inflammatory markers and treatment urgency may facilitate timely and accurate decision-making. These exploratory insights may help guide future screening protocol development for other pediatric inflammatory conditions requiring early immunosuppressive interventions. Implementing such considerations in clinical practice may help prevent diagnostic delays and optimize treatment decisions regarding children with acute inflammatory diseases. Although QFT may not serve as a definitive screening tool in acute KD, recognizing its diagnostic limitations and situations in which its results may be unreliable is critical for safe and timely clinical decision-making. Rather than validating QFT utility, our findings emphasize when its results should be interpreted with caution.

## Appendices

Excluded positive pediatric case

A single pediatric case with a positive QuantiFERON®-TB Gold Plus (QFT) result was excluded from the statistical analysis due to its distinct epidemiological and clinical background. The patient was a 56-month-old boy who had recently traveled from China and was later diagnosed with latent tuberculosis infection (LTBI) based on a positive QFT, epidemiological exposure to a known tuberculosis (TB) patient in China, and absence of clinical symptoms or radiographic findings consistent with active TB. The case also involved complex immunosuppressive therapy (infliximab, methylprednisolone, and cyclosporine A). Inclusion of this case would have introduced significant heterogeneity to the current cohort, which focused on indeterminate results in a relatively homogeneous domestic pediatric population. The clinical course of this case has been reported in detail elsewhere (Yokoyama et al., Cureus 2024; DOI: 10.7759/cureus.70407).
